# Medial posterior tibial slope measurements are overestimated on long radiographs and 3D CT compared to measurements on short lateral radiographs

**DOI:** 10.1002/jeo2.70120

**Published:** 2024-12-18

**Authors:** Ahmed Mabrouk, Arthur Chou, Wiemi Duouguih, Shintaro Onishi, Alfred Mansour, Matthieu Ollivier

**Affiliations:** ^1^ Department of Trauma & Orthopaedics Basingstoke and North Hampshire Hospital Basingstoke UK; ^2^ Department of Orthopaedics and Traumatology, Aix Marseille Univ, APHM, CNRS, ISM, Sainte‐Marguerite Hospital Institute for Locomotion Marseille France; ^3^ Department of Orthopaedic Surgery The Union Memorial Hospital Baltimore Maryland USA; ^4^ Department of Orthopedic Surgery, McGovern Medical School University of Texas Health Science Center at Houston Houston Texas USA

**Keywords:** 3D reconstructed CT, ACL injury, medial posterior tibial slope, posterior tibial slope

## Abstract

**Purpose:**

This study assessed the measurements of the medial posterior tibial slope (MPTS) using long radiographs and three‐dimensional (3D) computed tomography (CT) scans and compared them to measurements taken on short lateral knee radiographs. The study aimed to identify whether the at‐risk slope measurements previously defined on the short radiographs would be similar to long radiographs and 3D CT scans.

**Methods:**

A retrospective radiological review of 52 cases, who underwent planning for a slope‐changing high tibial osteotomy and had short and long lateral radiographs and 3D CT scans of the tibia. Two independent observers measured the MPTS on the three modalities. The MPTS was defined as the angle between a tangent to the medial tibial plateau and the referenced tibia anatomical axis. The MPTS measurements from the short and long radiographs were compared to each other and then were compared to the measurements performed on the CT scan. False positives were defined as those cases with MPTS measurements of >78° on CT scans or long radiographs while having measurements ≤78° on short radiographs. These false positive cases are the ones which would be falsely labelled as having an abnormal slope based on the previously validated short radiograph slope threshold ≥12°.

**Results:**

A total of 52 cases were analysed (67.9% males and 32.1% females). The mean age was 27 ± 5.4 years. The mean weight was 71.5 ± 7.7 kg, and the mean height was 1.8 ± 0.1 m. The mean MPTS measured on the short radiographs was 77.3 ± 2.3°; on the long radiographs, it was 75.8 ± 2.0°; and on the CT scan, it was 75.3 ± 2.1°. There was a positive correlation between the measurements taken on both the short and long radiographs (*r* = 0.9) (*p* < 0.001). Additionally, there was a positive correlation between CT tibial slope measurements and both short and long radiographs tibial slope measurements (*r* = 0.86, *r* = 0.87), respectively (both *p* < 0.001). False positives were 13 (25%) patients on long radiographs, and 12 (23.1%) patients on CT scans, who had their MPTS measurements ≤78° (equivalent of PTS ≥ 12°) while their measurements were >78° on the short radiographs.

**Conclusion:**

Measurements of the MPTS can be overestimated by 1.5–2° on long lateral knee radiographs or 3D CT scans compared to measurements taken on short lateral radiographs. Different thresholds for the abnormal PTS measurements on long radiographs and CT scans, should be defined, considering the overestimated measurements in these modalities.

**Level of Evidence:**

Level IV case series.

**Abbreviations**
3D: three‐dimensional
ACL: anterior cruciate ligament
ANOVA: analysis of variance
CT: computed tomography
HTO: high tibial osteotomy
MPTS: medial posterior tibial slope
MRI: magnetic resonance imaging
PTS: posterior tibial slope

## INTRODUCTION

In knee kinematics, the posterior tibial slope (PTS) contributes to the degree of tibial translation, the strain on either the native or grafted cruciate ligaments, and the pressure distribution on the cartilage [1, 2, 13, 15, 20, 22]. The PTS can be measured on either the medial or lateral tibial plateau, and the difference between the medial PTS (MPTS) and lateral PTS (LPTS) can affect dynamic landing knee biomechanics [23, 29]. Hence, precise measurements of both MPTS and LPTS could be valuable in screening the individuals at higher risk for cruciate ligament injury.

The PTS is identified by the angle between a tangent to the posterior inferior tibial plateau and the anatomic axis of the tibia [3], and averages approximately 80 ± 3°, for either the MPTS or LPTS on long leg radiographs [4, 9, 12]. There are multiple studies that investigated and validated methods for measuring PTS using either radiographs [4], computed tomography (CT) scan [19], or magnetic resonance imaging (MRI) [2, 15, 16, 17, 21, 29, 30, 31]. Measurements taken on these different imaging modalities have been reported to have no significant difference [32]. However, there are no studies that reported and validated measuring the MPTS on short radiographs compared to the measurements taken on long radiographs or CT scans.

This study presents measurements of the MPTS on different imaging modalities. It aimed to identify whether the at‐risk slope measurements previously defined on short radiographs will be similar on long radiographs and 3D CT Scans. It was hypothesized that there would be no difference in the MPTS measurements taken on long radiographs or the 3D CT scans compared to short radiographs.

## METHODS

After institutional review board approval (PADS24‐172_dgr), a retrospective review of 52 patients who underwent planning for a slope‐changing high tibial osteotomy (HTO) was undertaken.

### Inclusion and exclusion criteria

The study included patients with the three imaging modalities. Patients with deformities in the tibial shaft or distal tibia were excluded. Patients with reverse slopes in the presence of hardware or any evidence of previous bony surgery were also excluded. Cases with incomplete imaging profiles were also excluded.

### Imaging protocol

All patients had, short and long, lateral radiographs of the tibia and CT scan of the full‐length tibia. The short and long radiographs had 40 and 80 cm of the tibia imaged, respectively, and both were taken in 20° of knee flexion and neutral rotation with the femoral condyles overlapping as much as possible to ensure pure lateral. All radiographic imagings were performed by highly trained radiographers specializing in knee imaging.

### MPTS measurements technique

#### Radiographic measurements

**Figure 1:**
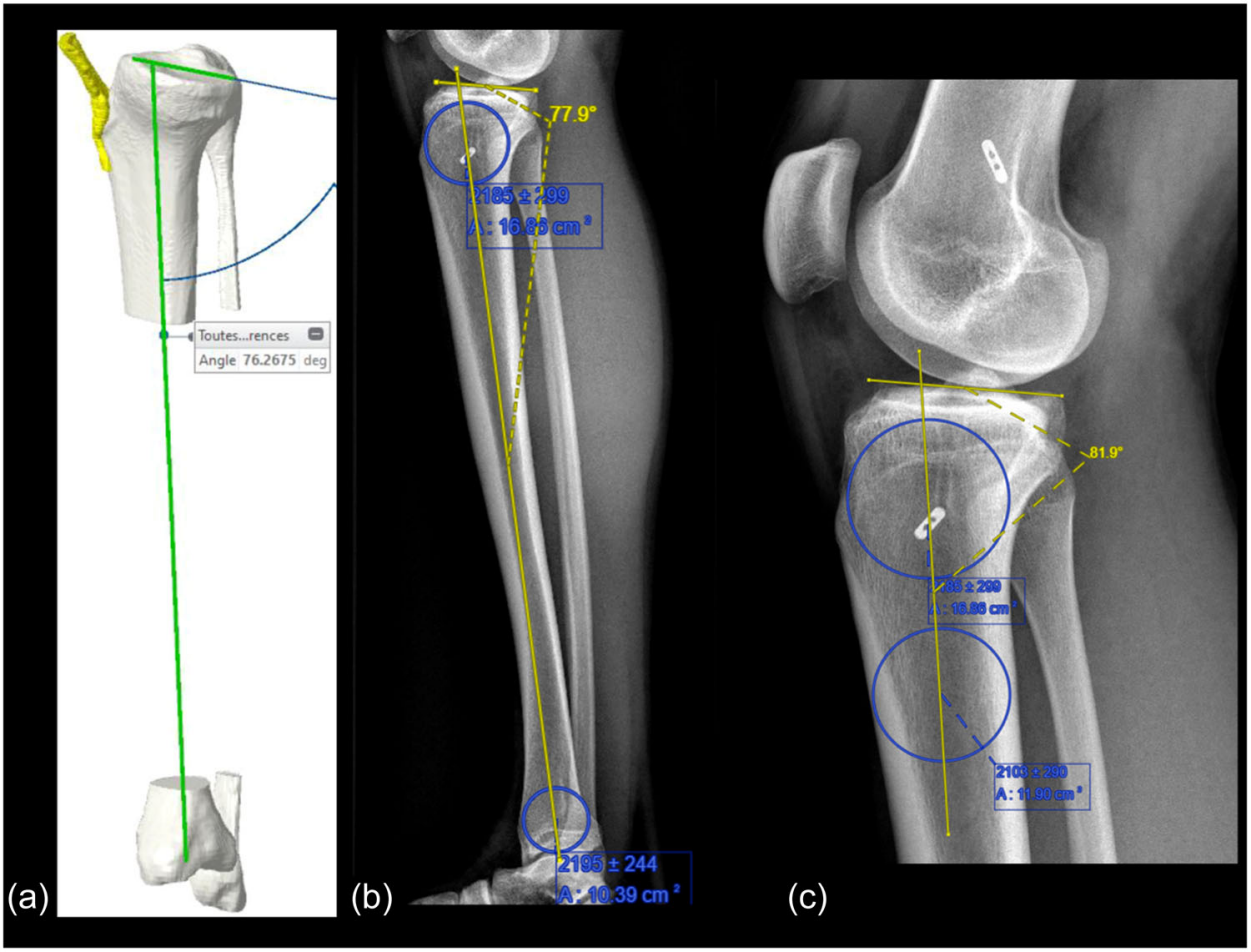
Demonstrates. (a) MPTS measurements on the 3D reconstructed CT images; (b) MPTS measurements on the long lateral radiograph (full‐length tibia); (c) MPTS measurement on the short lateral knee radiographs. 3D, three‐dimensional; CT, computed tomography; MPTS, medial posterior tibial slope.

Measurements of the radiographic MPTS were performed using PeekMed Software®. Two independent observers measured the MPTS on the three modalities. The anatomic tibia axis was defined as the mid‐diaphyseal line in the radiographs [26]. The tibia anatomic axis was identified on the three modalities using the circle method as described by Hudek et al. [16, 17]. This entails, in the short radiographs, drawing two circles to fit within the proximal, anterior and posterior cortices of the tibia. The distal circle was drawn so that it fits within the anterior and posterior cortices of the tibial diaphysis with its centre 20 cm from the tibial plateau. However, in the long radiographs, the method involved drawing two circles; one circle fits within the proximal, anterior and posterior cortices of the tibia, and the second circle fits within the distal, anterior and posterior cortices of the tibia. A line connecting the centres of the circles defined the tibia anatomic axis. The MPTS line was defined with a line tangent to the medial tibial plateau subchondral bone from the most anterior proximal point to the most posterior point. All measurements of the MPTS were identified as the angle between the MPTS line and the line of the tibia anatomic axis (Figure 1).

#### CT scans measurements

For the CT scans, a CT‐based 3D model of each tibia was created. These CT scans were obtained as part of the process of creating patient‐specific instrumentation (PSI) for the planned slope‐changing HTO [14]. The following protocol was applied for the CT scanning using Centricity MPR modelling System (GE): 2 mm slice (for hip and ankle), from ilium to foot and 0.625 mm slice around the knee (Newclip protocol spacing is between 0.625 and 1). Using this system, the pelvis, both femurs and both tibiae were examined. All measurements were calculated using an algorithm that identified landmarks on the corresponding bone, which created reproducible and consistent constructs for each case. Previous accuracy and reproducibility analysis estimated that this system allows automated measurements of upper femoral anatomy with a margin of error of <2 mm and <1° [10]. The tibia mechanical axis was defined from the knee centre to the ankle centre. The intersection of the medial tibial articular surface with the sagittal axis was established to determine the medial sagittal tibial axis. The posterior angle between the medial tibial sagittal axis and the tibial mechanical axis in the sagittal plane was identified as the MPTS angle.

The MPTS measurements from the short and long radiographs were compared to each other and then were compared to the measurements performed on the CT scan. False positives were defined as those cases with PTS measurements of >78° on CT scans or long radiographs, while having measurements of ≤78° on short radiographs (equivalent to PTS of ≥12°).

### Statistical analysis

Data were analysed with statistical software (R Core Team (2022) R Foundation for Statistical Computing, Vienna, Austria). Descriptive statistics for continuous variables were reported as means ± standard deviations [95% confidence intervals]. A Pearson correlation coefficient was computed to assess the linear relationship between CT tibial slope measurements and both short and long radiographs tibial slope measurements, as well as the linear relationship between the measurements on both the short and long radiographs. Analysis of variance (ANOVA) was conducted for inter‐modality measurements comparison and was followed by Tukey post hoc test. Scatterplots with regression lines were used to graphically represent the relationship between tibial slope measurements by the three modalities: CT, short radiographs and long radiographs. Inter‐ and intra‐rater reliability was assessed using intra‐class coefficients (ICCs). An ICC greater than 0.9 was considered excellent and ICC between 0.8 and 0.9 was considered good. A sample size of 52 patients based on a mean slope of 80 ± 2° in the presented series achieves a power of 80%.

## RESULTS

A total of 52 cases were analysed, including 67.9% males and 32.1% females. The mean age was 27.3 ± 6.2 years. Patient demographics are presented in Table 1.

**Table 1:** Patient demographics.

Variable	Value
Age (years)	27 ± 5.4 [25.6–28.5]
Weight (kg)	71.5 ± 7.7 [69.4–73.6]
Height (m)	1.8 ± 0.1 [1.8–1.8]
BMI (kg/m^2^)	22.5 ± 2.2 [21.9–23.1]
Gender	
Male	35 (67.3%)
Female	17 (32.7%)
Side	
Right	24 (46.2%)
Left	28 (53.8%)

Abbreviation: BMI: body mass index.

The mean MPTS measured on the short radiographs was 77.3 ± 2.3° [95% CI: 76.7–77.9], and on the long radiographs was 75.8 ± 2° [95% CI: 75.2–76.3] compared to the mean MPTS measured on the CT scan of 75.3 ± 2.1° [95% CI: 74.7–75.8]. Inter‐ and intra‐rater ICCs were 0.72 and 0.78, respectively (*p* < 0.001).

**Figure 2:**
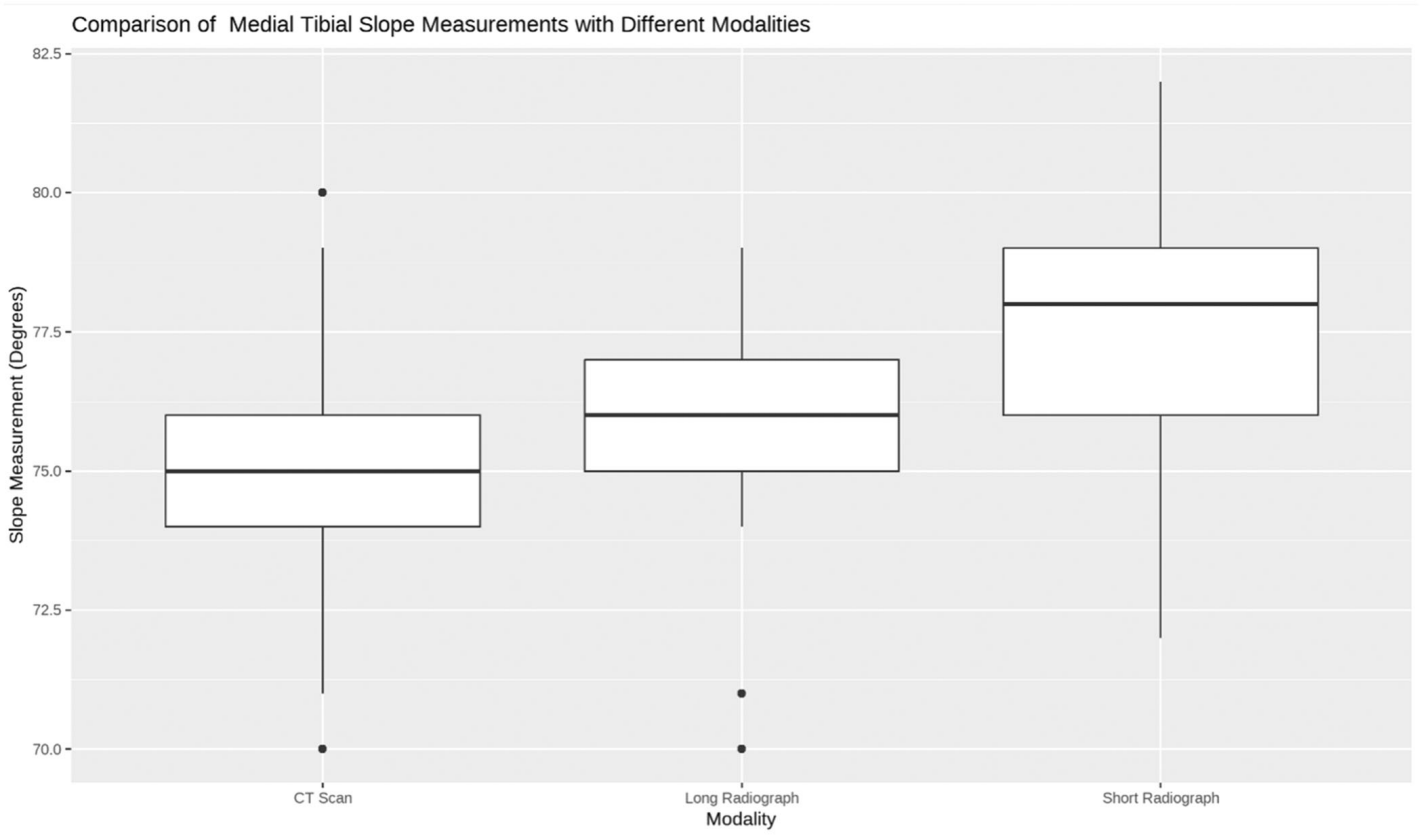
A boxplot diagram comparing the measurements from the three modalities.

Inter‐modality measurements ANOVA was significant (*p* < 0.001). Tukey post hoc test showed a significant difference between the measurements of CT scans and short radiographs of 2° (*p* < 0.001) and between the measurements of the long and short radiographs of 1.5° (*p* < 0.001). There was no significant difference between the measurements of the CT scans and long radiographs (*p* = 0.5) (Figure 2).

**Figure 3:**
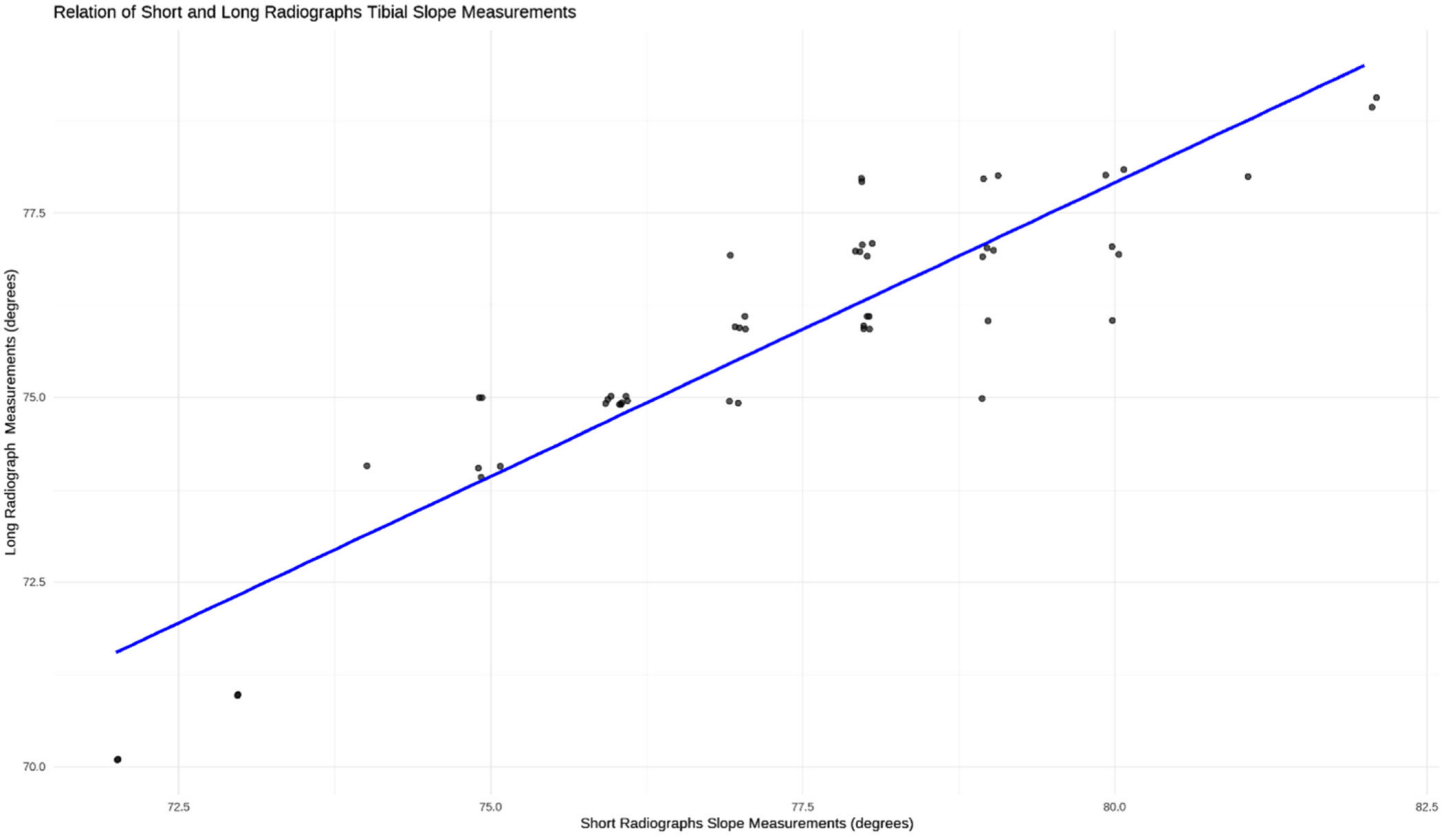
Scatterplot with linear regression line demonstrating the correlation between the tibial slope measurements taken on the long and short radiographs.

**Figure 4:**
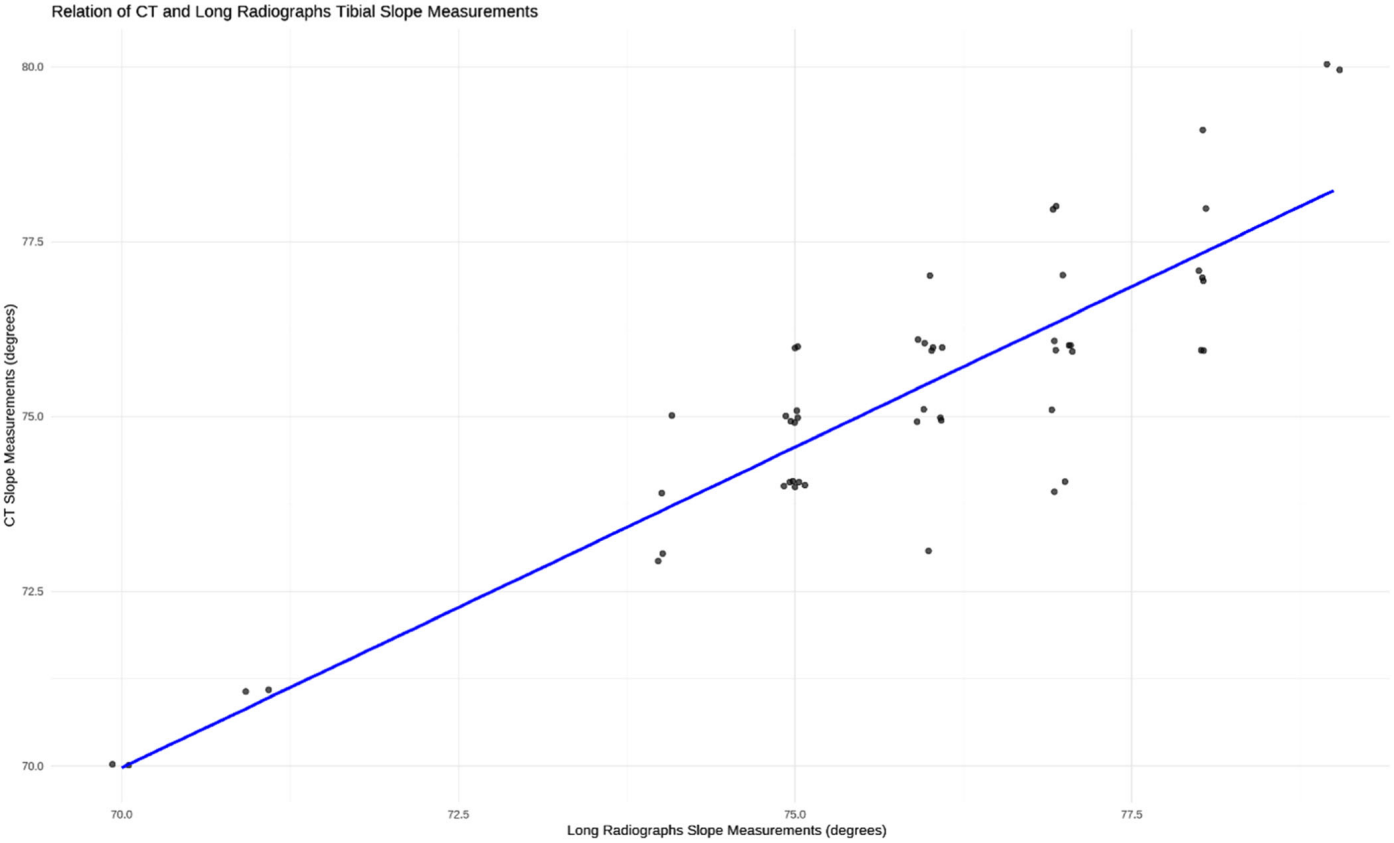
Scatterplot with linear regression line demonstrating the correlation between tibial slope measurements taken on the long radiographs and CT scan. CT, computed tomography.

**Figure 5:**
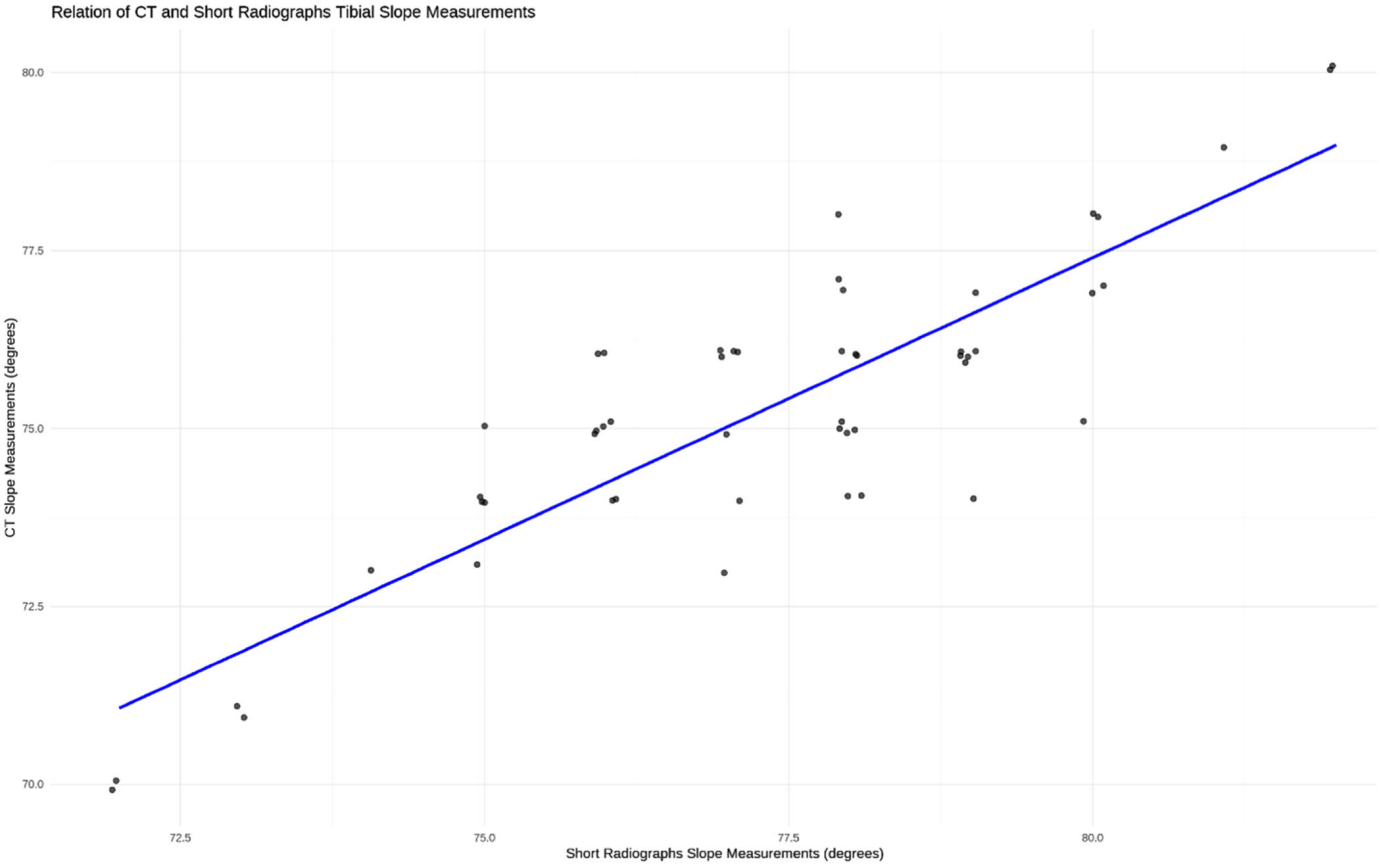
Scatterplot with linear regression line demonstrating the correlation between tibial slope measurements taken on the short radiographs and CT scan. CT, computed tomography.

There was a positive correlation between the measurements taken on both the short and long radiographs (*r* = 0.9) (*p* < 0.001). Additionally, there was a positive correlation between CT tibial slope measurements and both short and long radiographs tibial slope measurements (*r* = 0.86, *r* = 0.87), respectively (both *p* < 0.001) (Figures 3, 4, 5).

The total number of patients who had MPTS measurements of ≤78° (equivalent to PTS ≥ 12°) was on long radiographs (*n* = 50), on CT scans (n = 49) and on short radiographs (*n* = 37). The mean value of MPTS in those patients was 76.3 ± 1.8° on the short radiographs, 75.1 ± 1.9° on long radiographs (mean difference vs. short radiograph of 1.2°) and 74.6 ± 1.8° on CT scans (mean difference vs. short radiograph of 1.7°).

### False positives

False positives were 13 (25%) patients on long radiographs, and 12 (23.1%) patients on CT scans, who had their MPTS measurements ≤78° (equivalent of PTS ≥ 12°) while their measurements were >78° on the short radiographs.

## DISCUSSION

The most important finding in the presented study demonstrates that measurements of the MPTS can be overestimated by 1.5–2° when taken on either long lateral knee radiographs or 3D reconstructed CT scan images, compared to the measurements taken on the short lateral knee radiographs. Similar findings were reported by Ni et al. [25] with 1.8° overestimation in PTS measurements (average of MPTS and LPTS) taken on full‐length true lateral tibia radiographs compared to half‐length radiographs. Hence, using a PTS threshold of ≥12° as a risk factor for ACL or ACL‐graft failure, which was previously defined on short lateral knee radiographs [28, 34], could result in false positives when set as a threshold for measurements taken on CT scans and long radiographs.

The PTS has gained increased interest in recent years, as an excessive and insufficient slope can significantly alter the biomechanics of the knee [7]. An increased PTS has been demonstrated to be one of the risk factors for failure after anterior cruciate ligament (ACL) reconstruction, whereas a decreased PTS can lead to increased stresses on the posterior cruciate ligament and potentially recurvatum of the knee [8, 28, 35]. Nevertheless, the ideal method to assess the PTS remains inconclusive as each imaging modalities have different advantages and shortcomings [18, 19, 24, 36].

Conventional short lateral radiographs are frequently performed as a standard of practice for follow‐up after a variety of knee surgeries. It is a relatively reliable radiologic modality, and the normal value of the PTS has been described to be between 80° and 80.1° [9, 17]. However, radiographs are operator and position‐dependent, as both rotation and knee flexion angle have been shown to influence radiographic PTS measurements [18, 24, 33, 37]. Chen et al. [5] demonstrated that at approximately 40° of knee flexion, there is 11.4° of tibial internal rotation. Zhang et al. [37] noted that the PTS increased by 3° at 40° of tibial rotation. Faschingbauer et al. [11] noted an overestimation of the PTS by 3° when measured on a short lateral radiograph and concluded that the long‐leg lateral radiographs are more accurate. However, the presented study demonstrated an overestimation of MPTS measurements on long radiographs and 3D CT scans compared to short radiographs. On contrary, Dean et al. [7], measured the PTS of 140 patients and concluded no significant difference in PTS measurements referenced to the anatomic axis of the tibia [7]. Utzschneider et al. [32] described their method of assessing the PTS by obtaining the MPA, which is the mean of the two angles formed between the tibial plateau and the tibial axes referenced to both the anterior tibial cortex and posterior tibial cortex, on a short lateral radiograph. They concluded that PTS assessed with reference to the MPA was comparable to PTS measurements taken on both CT scans and MRI [32].

Webb et al. [34] and Salmon et al. [28] identified a PTS of ≥12° as the highest predictor of ACL injuries. However, this threshold was identified on measurements taken on short lateral radiographs. In the presented study, patients with MPTS of 76.3 ± 1.8° (equivalent to 13.7°) on the short lateral radiographs had their MPTS measurements on both long radiographs and CT scans as 75.1 ± 1.9° and 74.6 ± 1.8°, which is equivalent to 14.9° and 15.4°, respectively. This demonstrates the overestimation of MPTS measurements on the long lateral radiographs and CT scans compared to the measurements taken on the short radiographs. So, patients with lower slope values on short radiographs could be falsely identified as patients with at‐risk slopes if measured on long radiographs or CT scans, which could result in offering unindicated surgeries that potentially would have adverse outcomes. Consequently, the threshold for the at‐risk slope, on long radiographs and CT scans, needs to be further defined.

Measurements of PTS on CT scans can be performed either on serial cuts or 3D reconstructed images [27, 37]. A benchmark study reported, on CT‐based measurements, the mean global, MPTS and LPTS as 6.3° (range, −5.5° to 14.7°; 1% ≥12°), 6.2° (range, −4.1° to 17.2°; 3% ≥12°) and 5.3° (range, −4.7° to 16.2°; 2% ≥12°), respectively [27]. Kessler et al. [19] noted that PTS measurements on radiographs showed high variations based on tibial rotation with errors reported up to 14°. However, PTS measurements taken on multi‐sliced CT scans were more accurate with errors reported of 3° or less [19]. This discrepancy has been refuted by the findings in the presented study, with no significant differences in PTS measurements taken on short lateral radiographs versus 3D CT scans: 78.2 ± 4.0° versus 76.4 ± 4.6°, respectively. Moreover, CT scan is of a higher cost and exposes the patient to a higher radiation dose (3.0–8.5 mSv), and it has been reported that >10 mSv is associated with increased cancer risk [6].

Another commonly used modality in evaluating knee injuries and soft tissue structures is MRI [24]. Hudek et al. [17] described a reliable method to measure PTS on MRI, however, the mean PTS measurements on the MRI differed by 3.4° compared to the measurements taken on the lateral knee radiographs, 4.8 ± 2.4° versus 8.2 ± 2.8°, respectively. In addition to better reproducibility of the PTS measurements on the radiographs compared to the MRI scan [17]. This difference can be explained by the different MRI sequences used to measure the tibial slope, subchondral versus cartilage sensitive sequences, which can significantly change the PTS [18]. Therefore, the treating physician should interpret PTS measured on MRIs with caution and understand the type of sequence being applied [18].

There are some limitations to the presented study, including being a non‐controlled and non‐randomized study. In addition, all PTS measurements were done on patients presented with knee pain with no previous ACL reconstruction. Potentially, a cohort of patients with failure of ACL reconstruction would have better represented the desired population. Another limitation is that MRI scans were not performed, which could have further delineated cartilage and soft tissue factors that may affect the MPTS.

## CONCLUSION

Measurements of the MPTS can be overestimated by 1.5–2° on long lateral knee radiographs or 3D CT scans compared to measurements taken on short lateral radiographs. Different thresholds for the abnormal PTS measurements on long radiographs and CT scans, should be defined, considering the overestimated measurements in these modalities.

## AUTHOR CONTRIBUTIONS

The following authors, Ahmed Mabrouk, Arthur Chou, Wiemi Duouguih, Shintaro Onishi, Alfred Mansour and Matthieu Ollivier, have participated in the content and design of the study and have seen and agreed with the contents of the manuscript.

## CONFLICT OF INTEREST STATEMENT

The authors declare no conflicts of interest.

## ETHICS STATEMENT

Institutional review board approval (PADS24‐172_dgr) was obtained for this study.

## Data Availability

All data are available upon request with formal authorization from the hospital.
